# MWA Performed at 5.8 GHz through ‘Side Firing’ Approach: An Exploratory Study

**DOI:** 10.3390/s22239320

**Published:** 2022-11-30

**Authors:** Anna Bottiglieri, Christopher Brace, Martin O’Halloran, Laura Farina

**Affiliations:** 1 Electrical and Electronic Engineering, National University of Ireland Galway, H91 TK33 Galway, Ireland; 2 Translational Medical Device Lab, National University of Ireland Galway, H91 TK33 Galway, Ireland; 3 Department of Biomedical Engineering, University of Wisconsin-Madison, 1415 Engineering Drive, Madison, WI 53706, USA; 4 Department of Radiology, University of Wisconsin-Madison, 1111 Highland Ave, Madison, WI 53705, USA

**Keywords:** microwave ablation, directional heating, operating frequency

## Abstract

Recent studies have shown that ablation techniques have the potential to eradicate adrenal adenomas while preserving the functionalities of the adrenal gland and the surrounding anatomical structures. This study explores a new microwave ablation (MWA) approach operating at 5.8 GHz and using anatomical and dielectric characteristics of the target tissue to create directional heating patterns. Numerical simulations are executed in planar and 3D adrenal models, considering two energy doses. The numerical study is refined accounting for the vaporization of the tissue water content. *Ex vivo* experimental evaluations on porcine adrenal models complete the study. The numerical and experimental results show that spherical ablation zones are able to cover the target for both energy doses considered. Nonetheless, most of the non-targeted tissues can be preserved from excessive heating when low energy level is used. Numerical models accounting for water vaporization are capable to foresee the experimental temperature values. This study shows that the proposed MWA directional approach operating at 5.8 GHz can be considered for creating effective and selective ablation zones.

## 1. Introduction

Microwave thermal ablation (MWA) is a minimally invasive technique to selectively target and destroy biological tissues. In recent years, MWA has been explored to treat small adrenal adenomas (≤20 mm diameter) in patients who are not surgical candidates [1,2,3,4]. A ‘side firing’ approach has been proposed to direct energy more specifically into the tumor, which may overcome risks linked to the position of adrenal adenomas in proximity to kidney, large and medium blood vessels, or nerves [5,6]. Alternatively, a method that leverages differences in dielectric properties between the adrenal medulla and cortex to enhance heating of the tumor has been explored [7,8,9]. The fat layer surrounding the adrenal glands has been used to confine the electromagnetic field and create asymmetric ablation profiles with an omnidirectional antenna at 2.45 GHz. 

Contrast between the dielectric properties of the tissues of interest increases with frequency [10,11]. For example, the contrast in effective conductivity between the adrenal cortex and fat is nearly two-fold higher at 5.8 GHz as compared with the contrast at 2.45 GHz. This higher contrast in the effective conductivity obtained at 5.8 GHz presents an opportunity to improve the deposition of the EM energy in the target tissue. 

In this work:

- A numerical study is conducted using a triaxial monopole applicator operating at 5.8 GHz. The applicator is placed parallel to the interface between the adrenal cortex and fat tissue. Two different orientations of the antenna are considered with reference to the interface between the two tissues. The distributions of SAR and temperature in simplified multilayered and 3D models are investigated.
- *Ex vivo* porcine adrenal tissues are used to validate the numerical study. The ablation profiles and the increase in temperature are monitored and compared with the numerical results.
- The influence of water vaporization on the distribution of temperatures in the tissue target is discussed.
- The structure of this paper is as follows: In Section 2, we describe the numerical models specifying the properties of the adrenal tissues at 5.8 GHz and the experimental setup; in Section 3, we show and discuss the ablation profiles obtained for each numerical geometry, the temperature values as a function of the radial distance from the applicator and the time, and the effect of water vaporization on the temperatures achieved in the adrenal tissues; in Section 4, the conclusions are presented.

## 2. Materials and Methods

### 2.1. Numerical Study

#### 2.1.1. Geometrical Models

**Figure 1:**
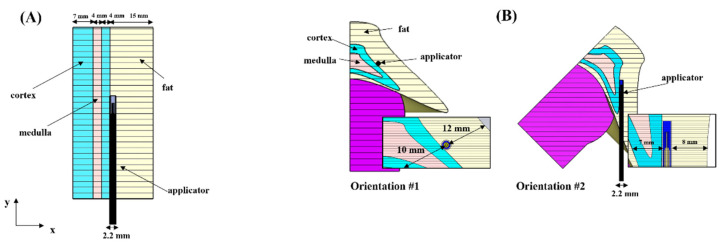
(**A**) Simplified geometry for modeling each functional tissue of the adrenal gland and surrounding fat layer; (**B**) 3D geometrical models of the adrenal tissues and the adjacent fat layer. The triaxial MW applicator optimized to operate at 5.8 GHz was placed parallel to the interface between fat and the adrenal cortex.

Figure 1A depicts a multilayer geometrical drawing to model the adrenal tissues and the adjacent fat layer. The multilayer structure—four 3D domains of 30 mm in height, 30 mm in thickness and varying widths—is a simplified representation of the adrenal gland. In the model, the medulla was enclosed within two layers named ‘outer cortex’ (adjacent to the fat layer) and ‘inner cortex’ (far from the fat layer) representing the adrenal cortex.

Depending on the type of tissue, different widths were assigned to each layer: 15 mm for fat, 4 mm both for the inner part of the cortex and the medulla, 7 mm for the outer layer of cortex. The width of the fat layer was set equal to 15 mm. A previous study [9] showed comparable asymmetric ablation profiles for different widths of the fat layer (15, 10, and 5 mm). The width of the medulla and the inner layer of cortex represent a realistic margin between the two functional adrenal tissues [12,13,14]. The width of the outer layer of cortex was set at 7 mm to minimize the impact of the exterior boundaries. Figure 1B shows a three-dimension model used to study the influence of the orientation of the MW applicator on the ablation profiles at 5.8 GHz. The dimensions of the model and the orientations of the MW applicator with respect to the cortex-fat interface are consistent with those reported in [9].

Table 1 lists the dielectric and thermal properties assigned to each layer, representing the adrenal tissues (i.e., medulla and cortex) and the external fat layer. The values of relative permittivity and effective conductivity described in [11] were assigned to the adrenal cortex and medulla. The dielectric properties assigned to the fat tissue and the thermal properties of each tissue were acquired from [15]. The dielectric properties of air ($\varepsilon_{r}=1,\;\sigma =0$ S·m^−1^) were assigned as the background material, which is representative of the *ex vivo* conditions of the experimental scenario. 

The triaxial monopole applicator was placed parallel to the interface between the fat and the adrenal cortex. The antenna was operated at 30 W and 60 W for 60 s. The applicator was designed as described in [16]. The overall diameter of the applicator is 2 mm. The antenna was tuned to radiate at 5.8 GHz by reducing the length of the radiating element to 3 mm.

The minimum requirement of at least 10 cells per wavelength model was upheld. A total of 3,003,520 tetrahedral meshing cells ranging between 0.2 mm and 3.3 mm in size discretized the entire planar model. For the 3D model, only the right half side of the geometry was considered, to reduce the computational load; a total of 34,776,336 meshing cells ranging between 0.02 mm and 0.5 mm discretized the geometry. 

#### 2.1.2. Electromagnetic and Thermal Simulations

The thermal transient solver included in the full-wave electromagnetic software (CST MWS Suite 2018, Darmstadt, Germany) was used to solve the Helmholtz’s equation, reported in Equation (1),

(1) $$\nabla^{2}E-k_{0}^{2}\left(\varepsilon_{r}-\frac{j\sigma }{\omega \varepsilon_{0}}\right)E=0,$$

where E (V·m^−1^) is the electric field vector, $k_{0}$ (m^−1^) is the propagation constant in the free space, ω (rad·s^−1^) is the angular frequency, $\varepsilon_{0}$ is the relative permittivity in free space, $\varepsilon_{r}$ and σ (S·m^−1^) are the relative permittivity and the effective conductivity of the tissue.

Constant values of relative permittivity and effective conductivity were used in this study, because of the lack in models describing temperature-dependent changes in the dielectric properties occurring at 5.8 GHz. 

Then, the distribution of the specific absorption rate (SAR) and the temperature was calculated according to Equation (2),

(2) $$\mathrm{SAR}=\frac{\sigma \;{\left|E\right|}^{2}}{2\rho }\;,$$

where ${\left|E\right|}^{2}$ is the intensity of the electric field vector E and ρ (kg·m^−3^) is the density of the tissue. Scattering boundary conditions were applied to the outer boundaries of the simulation domain.

Once SAR is known, the increase in temperature can be determined solving the bioheat equation (BHE) described by Equation (3),

(3) $$\rho c\frac{\partial T}{\partial t}=\nabla \cdot \left(k\nabla T\right)+\rho SAR+\rho Q-m_{b}\rho \rho_{b}c_{b}\left(T-T_{b}\right),$$

where *T* (°C) is the temperature, *t* (s) is the time, c (J·kg^−1^·°C^−1^) is the specific heat capacity of the biological tissue, k (W·°C^−1^·m^−1^) is the thermal conductivity, Q (W·kg^−1^) indicates the metabolic heat generation rate, $\rho_{b}$ (kg m^−3^) is the density of blood, $m_{b}\cdot \rho_{b}$ (s^−1^) is the blood mass perfusion rate, $c_{b}$ (J·kg^−1^·°C^−1^) is the specific heat capacity of the blood, and $T_{b}$ (°C) is the temperature of the blood.

The terms indicating the blood perfusion ($m_{b}\rho \rho_{b}c_{b}\left(T-T_{b}\right)$) and the metabolic heat (ρQ) were excluded to better represent the *ex vivo* experimental conditions. 

Convective boundary conditions completed the bioheat equations. The convection coefficient (h) was assumed equal to 1000 (W m^−2^ °C^−1^) to model forced convection between the outer surface of the applicator and the surrounding tissue due to the cooling system. The temperature of the refrigerating water was set at 18 °C. Free convection was modeled between the outer surface of the tissue model and the surrounding environment assuming h = 5 (W m^−2^ °C^−1^). The temperature of the environment was set equal to 20 °C. 

Next, a second set of numerical simulations was executed to account for losses in the water content of the tissue due to vaporization. For these numerical simulations, the specific heat capacity c (J·kg^−1^·°C^−1^) in Equation (3) was modified according to the model developed by [17] and reported in Equation (4),

(4) $$c^{\prime }=c-\frac{\alpha }{\rho }\frac{\partial W}{\partial T},$$

where *α* indicates the water latent heat constant which is equal to 2260 (kJ·kg^−1^) and *W* is the percentage of water content. The changes in the water content of the tissue (*W*) as a function of the temperature are reported in Equation (5), in agreement with [18]:

(5) $$\{\begin{array}{c} 0.778\cdot \left(1-e^{\frac{T-106}{3.42}}\right)\;\;\;\;\;\;\;\;\;70\;{}^{\circ}C\leq T<100\;{}^{\circ}C\\ 7.053-0.064096\cdot T\;\;\;\;\;\;100\;{}^{\circ}C\leq T<104\;{}^{\circ}C\\ 0.778\cdot e^{-\frac{T-80}{34.37}}\;\;\;\;\;\;\;\;\;\;\;\;\;\;\;\;\;\;\;\;\;\;\;\;\;\;\;\;\;\;\;\;\;\;\;\;T\geq 104\;{}^{\circ}C. \end{array}\;$$

Equation (4) combined with Equation (5) describes the variation in the heat specific capacity linked to the loss of the tissue water content when temperatures exceed 70 °C. The model was originally developed accounting for the characteristics of the liver and the percentage of water content at the baseline conditions, which is about 80% of the mass of the tissue. In this study, the same model was adopted for the adrenal tissues, as a water content similar to liver is expected (70–76%) [19]. The model was not applied to the fat tissue which is characterised by a water content approximately four times lower than the adrenal gland and liver [19].

The derivative of water content (W) of the adrenal tissues as a function of the temperature was reproduced in MATLAB (R2017a, The MathWorks, Inc., Natick, MA, USA). Then, the values of the modified specific heat capacity were obtained for each temperature included in the range between 25 °C and 200 °C. The sequence of values at the corresponding temperature was loaded in CST MW Studio for both adrenal cortex and medulla. Then, bi-directional numerical simulations were enabled. The bi-directional simulations involved a continuous update of the heat specific capacity of the tissue with the temperature reached at a specific time. Thus, the temperature increment at the next time step was calculated based on the new value of specific heat capacity calculated at the related temperature. The update was performed at a time step of 1 s for the entire duration of the signal excitation. 

Because of the excessive computational load required for the 3D geometry of the adrenal gland, the vaporization model was considered only for the simplified geometry. The values obtained from each simulation were exported and analyzed in MATLAB.

For this study, standard simulations required approximately 7 h as compared with 19 h for the dynamic simulations, using the CST MWS Suite 2018 on an Intel^®^ Core^TM^ i7@ 4 GHz with 16 GB RAM.

### 2.2. Experimental Study

**Figure 2:**
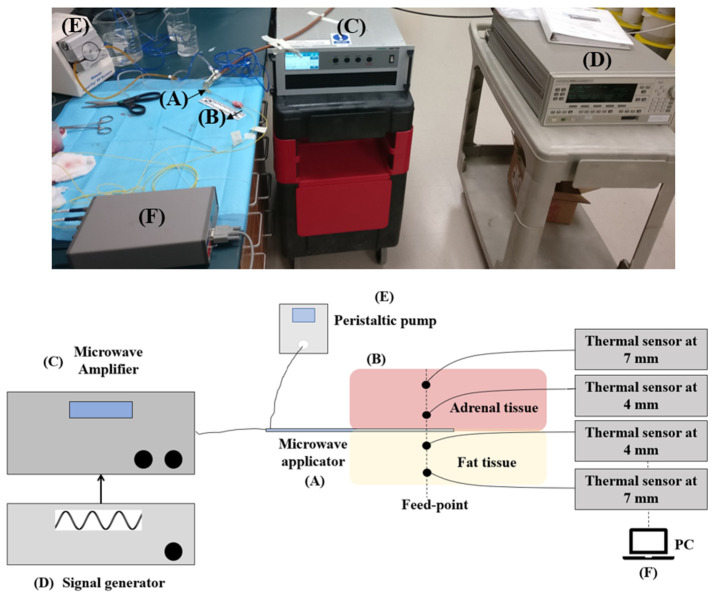
Setup adopted for the experimental validation of the computational results (**top**) and related schematization (**bottom**). The setup includes the microwave applicator (**A**) placed in the tissue (**B**) and powered for 60 s at the input power provided by the microwave amplifier (**C**) which amplifies the signal at the designed operating frequency (**D**); a peristaltic pump (**E**) connected to the inflow and outflow tubes of the refrigerating system integrated in the applicator; four fiber optic sensors connected to a laptop (**F**) to acquire values of temperature at 0.1 s time step.

The experimental study was conducted in porcine adrenal glands (*n* = 6) of 26.2 ± 1.0 (length), 15.3 ± 1.0 mm (width), 3.4 ± 1 mm (thickness), which were obtained from an abattoir. Values of relative permittivity and effective conductivity of the porcine model are comparable to those reported in sheep at 5.8 GHz [20]. Figure 2 provides an overview of the experimental setup adopted, along with the schematization of each component included in the setup. The applicator was positioned on the surface of the adrenal sample and covered by the surrounding fat layer. A signal generator (HP 83620A, 10 MHz–20 GHz, 15 dBm maximum output power) was used to provide a 10 dBm continuous wave sinusoidal signal at 5.8 GHz. A solid-state microwave amplifier AS0860-100D-, 0.8–6.0 GHz, 47 dB maximum gain (Milmega, Ametek, Ryde, UK) was used to amplify the signal to 34 W and 68 W, by setting the gain at 75% and 82%, respectively. The two different gains selected at the amplifier accounted for the power losses along to cable. Thus, the two values for power gain provided the required levels of power (30 W and 60 W) at the feed of the antenna. Water at a temperature of 17.2 ± 3.3 °C was circulated through the applicator by a peristaltic pump (Cool-tip RF System, Valleylab, Boulder, CO, USA). 

For each experiment, the temperature was monitored using four fiber optic sensors (Neoptix Inc., Québec, CA, USA). The fiber optic sensors were placed at a radial distance of approximately 4 mm and 7 mm from the antenna feed both in the adrenal tissue and fat tissue. These distances were selected in order to consider the increase in the temperature caused by the direct heating in proximity to the antenna (i.e., 4 mm) and the possible effects of the thermal diffusivity at higher radial distances from the feed (i.e., 7 mm). Because of the changes undergone by the sample at high temperatures (e.g., tissue contraction [21,22]), and the movement of the fiber optic sensors in the tissues, a shift in the starting position of the sensors may occur during the procedure. Table 2 lists the actual positions measured at the end of the MWA procedure for all the samples used.

For each combination of power and time setting (30 W–60 s and 60 W–60 s), ablation experiments were performed three times on a total of six samples. 

## 3. Results and Discussion

### 3.1. Numerical Study

**Figure 3:**
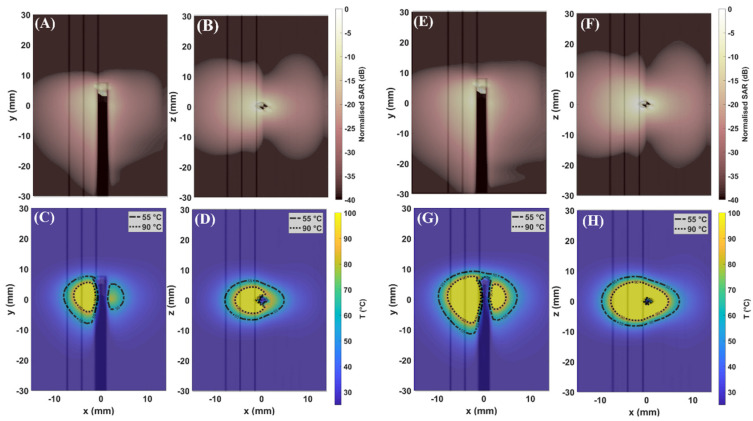
Maps of SAR: (**A**,**E**) (xy–plane); (**B**,**F**) (xz–plane) and maps of temperature: (**C**,**G**) (xy–plane); (**D**,**H**) (xz–plane), obtained in simplified model at 30 W and 60 W at 5.8 GHz. The applicator is aligned at the interface between fat and adrenal gland (Figure 1A). The longitudinal axis of the applicator is parallel to the y–axis.

**Figure 4:**
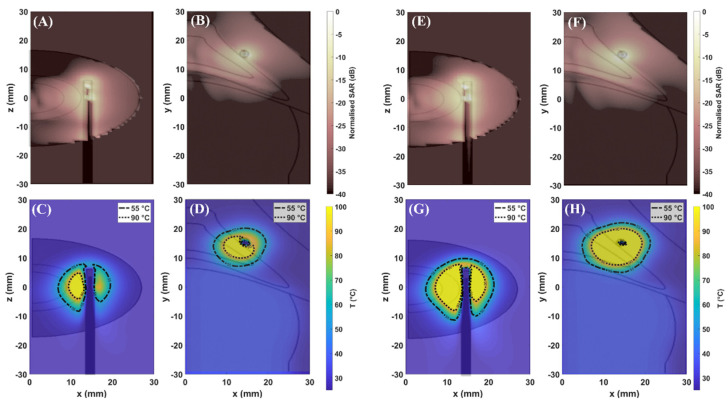
Maps of SAR: (**A**,**E**) axial plane (xz–plane); (**B**,**F**) coronal plane (xy–plane) and maps of temperature (**C**,**G**) axial plane (xz–plane); (**D**,**H**) coronal plane (xy–plane), obtained in the 3D model at 30 W and 60 W at 5.8 GHz. The applicator is aligned at the interface between fat and adrenal gland orientating the longitudinal axis parallel to the z–axis of the reference system (Figure 1B Orientation #1). Axial plane (xz–plane) and coronal plane (xy–plane) are indicated with reference to the axis of the antenna.

**Figure 5:**
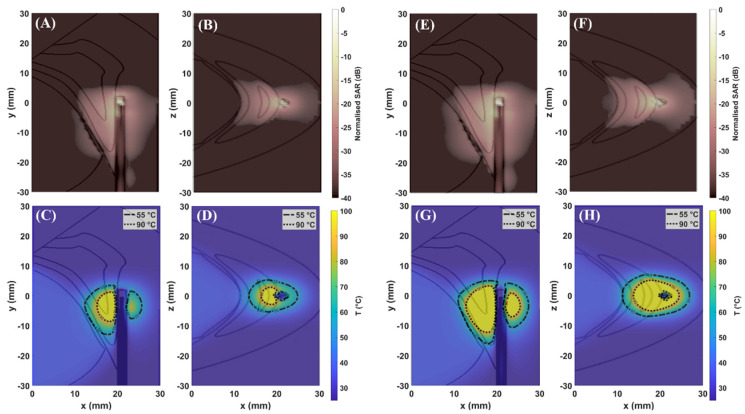
Maps of SAR: (**A**,**E**) axial plane (xy–plane); (**B**,**F**) coronal plane (xz–plane) and maps of temperature (**C**,**G**) axial plane (xy–plane); (**D**,**H**) coronal plane (xz–plane), obtained in the 3D model at 30 W and 60 W at 5.8 GHz. The applicator is aligned at the interface between fat and adrenal gland orientating the longitudinal axis parallel to the y-axis of the reference system (Figure 1B Orientation #2). Frontal plane (xy–plane) and coronal plane (xz–plane) are indicated with reference to the longitudinal axis of the antenna.

Figure 3 shows the distributions of SAR (first row) and temperature (second row) achieved in the simplified model at 30 W (A–D) and 60 W (E–H) for 60 s. Figure 4 and Figure 5 show the distributions of SAR (first row) and temperature (second row) obtained at 30 W–60 s and 60 W–60 s. The figures refer to the two different orientations of the antenna with respect to the interface between adrenal tissue and fat layer (Figure 1B). SAR and thermal distributions are provided for the axial and coronal planes. SAR values are normalized to the maximum and expressed in decibels (dB). 

Figure 3, Figure 4 and Figure 5 (first row) show that the SAR values higher than −20 dB are distributed over larger areas of the adrenal tissues as compared with fat. Thus, a difference in the absorption of the electromagnetic power between fat and adrenal tissues can be observed. As already observed in [7,8,9], the difference in the absorbed electromagnetic power between the two tissues results in asymmetric ablation profiles delineated by the isothermal contour at 55 °C (Figure 3, Figure 4 and Figure 5 (second row)). 

The extent of the ablation zones achieved in each tissue and for each setting are reported in Table 3. The influence of the higher values of effective conductivity both for adrenal gland ($\sigma_{cortex}$= 4.2 S·m^−1^) and fat ($\sigma_{fat}$ = 0.5 S·m^−1^) at 5.8 GHz as compared with the values at 2.45 GHz [9] ($\sigma_{cortex}$ = 1.6 S·m^−1^; $\sigma_{fat}$= 0.1 S·m^−1^) is visible. The higher contrast in effective conductivity ($\sigma_{cortex}$ − $\sigma_{fat}$ = 3.7 S·m^−1^) at 5.8 GHz results in higher heating rate as compared with those achieved at 2.45 GHz [9], using the same power and time settings. As a result, most of the electromagnetic field is absorbed close to the applicator, limiting the propagation of the field through the tissue. This result is linked to the difference in the penetration depth of the electric field which is lower at 5.8 GHz as compared with 2.45 GHz [23]. Asymmetric ablation profiles are visible, particularly in the case of low input power. In the simplified model, the ablation zones in the fat along the radial and longitudinal dimensions are 55% and 47% (30 W) and 30% and 25% (60 W) smaller, respectively, than in adrenal gland. In the 3D model, the radial extents of the ablation zone obtained at 30 W in the fat tissue are 38% and 44% smaller than in the adrenal gland for Orientation #1 and Orientation #2, respectively. At 60 W the ablation zone in fat becomes larger as compared with the case of low energy dose, but it is still 30% smaller than in the adrenal gland for both orientations. 

Table 3 highlights similar dimensions in the ablation zone between the two orientations, especially along the radial direction. The main difference between the two orientations is observed in the longitudinal dimensions which are approximately 25% smaller in the adrenal gland for Orientation #1 as compared with Orientation #2. This difference is linked to the difference in the geometrical characteristics of the two tissues, depending on the orientation of the MW applicator, as already observed at 2.45 GHz [9]. Overall, it should be noted that the 3D geometry has a smaller impact on the growth of the ablation zones as compared with 2.45 GHz [9].

The numerical results show that fat can provide a shielding effect on the ablation zone at 5.8 GHz. This effect was observed for both input powers, 30 W and 60 W. As already observed in the previous study [9], the setting at low input power allows for better control of the increase in temperature in both tissues. It is conceivable that low input power in combination with relatively short durations (60 s) helps to mitigate the effect of the heat transfer due to thermal conduction and yields ablation zones of smaller diameter. This effect is evident in fat, where the ablation zone is confined within 5 mm from the axis of the applicator. An ablation zone not exceeding 5 mm diameter in fat is desirable to protect the anatomical structures surrounding the adrenal glands from the risk of thermal damage. These sensitive anatomical structures including the inferior vena cava, abdominal aorta, diaphragm, and liver are a distance of 10–30 mm from the external surface of the periadrenal fat capsule, which is approximately 10–15 mm in thickness [24,25,26]. 

In addition, an improvement in the sphericity of the ablation profile can be noted in the adrenal tissue at 5.8 GHz both at 30 W and 60 W as compared with the ablation profiles achieved at 2.45 GHz [5]. The triaxial structure of the MW applicator used in this study partially enhances the ablation profile. The third metallic axis limits the effect of the backward currents flowing along the feedline of the applicator. However, the highest contribution in the sphericity of the ablation zone derives from the higher operating frequency used. An increase in the operating frequency requires a radiating element of shorter length [24,25]. Furthermore, at 5.8 GHz, the wavelength in the adrenal and fat tissues is 8 mm and 18 mm as compared with 18 mm and 41 mm at 2.45 GHz. Because of the shorter wavelength, at 5.8 GHz, the size of the meshing cells decreases by a factor of 10 as compared with 2.45 GHz. Thus, the geometry within each cell can be locally assumed as planar. As already observed, the 3D geometry has a smaller influence on the shape and size of the ablation zone as compared with the scenario at 2.45 GHz. Given that, the simplified model can be assumed to be a good representation of the tissue target, the advantages of the planar model as compared with the 3D model are: (1) the reduced computational load and (2) the negligible difference in the results between the two models. In particular, the multilayered geometry was used for investigating the influence of water vaporization on the ablation zone. 

Vaporization phenomena are linked to the high temperatures reached in the adrenal tissue, due to the faster interactions between the electromagnetic field and the tissue at 5.8 GHz. Thus, water vaporization is more influential at 5.8 GHz as compared with 2.45 GHz and needs to be considered in the numerical models. These numerical results are discussed along with the experimental data in the next sections.

### 3.2. Experimental Ex Vivo Assessment

#### 3.2.1. Ablation Zone and Temperature Increments

A total of six MWA procedures were carried out on six (*n* = 6) *ex vivo* porcine adrenal glands. Three adrenal glands were used for each setting, i.e., 30 W–60 s and 60 W–60 s. 

**Figure 6:**
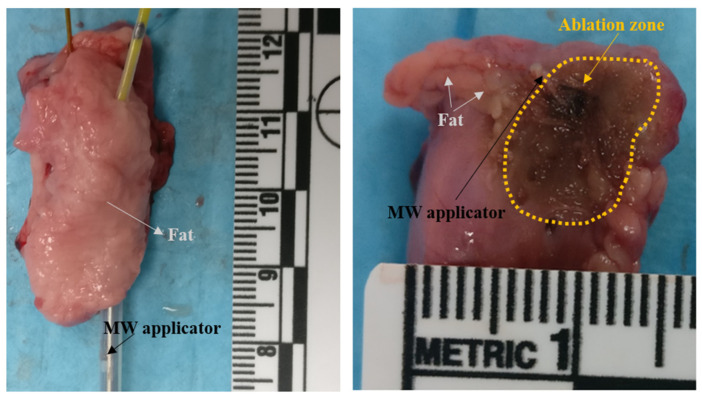
Section of the ablation zone obtained *ex vivo* in a porcine adrenal gland: The triaxial monopole MW applicator is placed on the surface of the gland and covered by fat, then it is excited at 5.8 GHz. The ablation zone in adrenal gland is marked (not visible in fat).

Figure 6 shows an example of the ablation zone obtained in the adrenal gland. The MW applicator was placed at the interface between the adrenal gland and the external fat layer to apply the ‘side firing’ approach. The ablation zone was evaluated by visual inspection on the surface of the adrenal cortex. Dark-colored areas delineated the ablation zones at 5.8 GHz instead of the whitening effect observed in the ablated area at 2.45 GHz [9]. This difference confirms the effect of the higher temperatures occurring in proximity to the applicator in the case of a higher operating frequency [4]. 

Table 3 reports the mean and standard deviation values of the radial and longitudinal dimensions of the ablation zone. The two dimensions were measured on each adrenal gland at the end of the procedure (60 s). The radial dimensions correlated with the radial values predicted by the numerical simulations in both adrenal models (planar and 3D) and for both input power levels (30 W and 60 W). The highest discrepancy was observed in the longitudinal dimensions. Visibly shorter ablation zones were observed in the experimental assessment as compared with the numerical findings. Thus, more spherical ablation zones were found in the experimental test. A similar finding was also observed at 2.45 GHz [13]. This discrepancy can be attributed to multiple factors including the actual shape of the sample, the changes in the structure of the tissue due to high temperatures, and the assumptions made about the tissue properties and their changes during the heating [21,22,27,28]. 

A recurrent swelling of the tissue was observed during the MWA procedures at 5.8 GHz, but not at 2.45 GHz [14]. This effect is likely linked to the high temperatures reached and the consequent increase in pressure developed inside the tissue due to vaporization [29].

#### 3.2.2. Effect of the Water Vaporization on Temperature Increments

**Figure 7:**
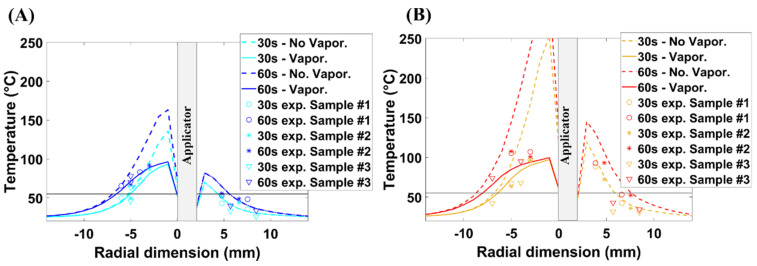
Numerical and experimental values of temperature along the radial distance from the MW antenna axis (applicator) both in the fat and the adrenal gland at 30 s and 60 s for 30 W (**A**) and 60 W (**B**). Numerical values include the temperature obtained using the standard BHE (dot lines) and the BHE modified to account for the effect of water vaporization (solid lines). Horizontal line highlights the temperature threshold at 55 °C.

Figure 7 shows the temperature values acquired experimentally in the adrenal gland and fat. The experimental values align well with the actual positions of the thermal sensors in the tissue, as reported in Table 2. The temperatures refer to two power levels, 30 W (A) and 60 W (B), and to two time steps, 30 s and 60 s. The curves of temperature derive from two different numerical scenarios: (1) without considering the effect of the vaporization model and (2) accounting for the vaporization phenomena. The experimental and numerical results are b oth expressed as a function of the radial distance from the longitudinal axis of the MW applicator. 

**Figure 8:**
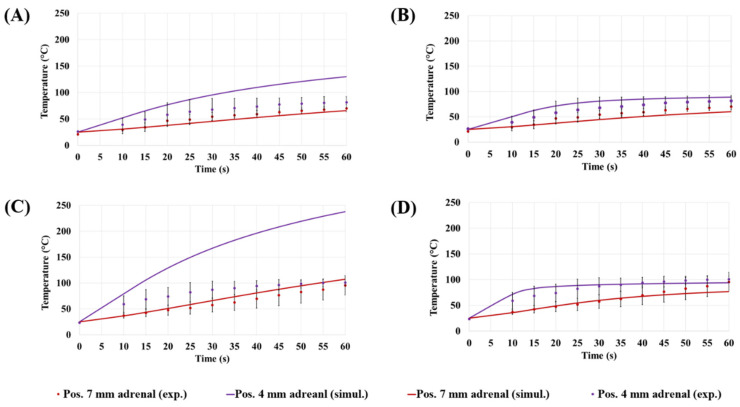
Comparison between the numerical results (solid lines) obtained in the case of standard BHE (**A**,**B**) and implementing the model of water vaporization (**C**,**D**). The increments of temperature were considered for two input powers: 30 W (**A**,**B**) and 60 W (**C**,**D**) and at distances from the MW applicator equal to 4 mm and 7 mm. Mean and standard deviation of the temperature values measured with fiber optic sensors were also reported (markers).

Figure 8 compares the experimental and numerical temperature increments as a function of time. In one case, the effect of water vaporization was neglected (A,C), and in the other case, the water vaporization was considered (B,D). Numerical and experimental values refer to 30 W (A,B) and at 60 W (C,D). Two different distances from the antenna feed were considered (4 mm and 7 mm), according to the starting positions of the thermal sensors. The mean values and standard deviation of the experimental measurements were reported. 

Figure 7 and Figure 8 show good correlations between the experimental and the numerical results, when the effect of vaporization is considered. The absence of water vaporization in the numerical simulations results in unrealistically high temperatures in proximity to the MW applicator. At 30 W, the temperature exceeds 120 °C after 30 s (Figure 7A). At 60 W, temperatures higher than 250 °C are observed after 30 s (Figure 7B). These results show that the standard BHE is not capable of accurately predicting the increase in temperature observed experimentally. This limitation results in a high discrepancy between the numerical and experimental data (Figure 8A,C). At 4 mm from the applicator, the measured values differ from the numerical findings by approximately 48 °C at 30 W (Figure 8A) and 137 °C at 60 W (Figure 8C). Further from the applicator (7 mm), the difference between the numerical and experimental data is within the standard deviation of the experimental data. 

Implementing the vaporization model, the maximum temperature reached is approximately 100 °C, which was observed in the case of the highest power and at the closest distance from the applicator (Figure 8D). The modified specific heat capacity described by Equation (4) accounts for the energy (i.e., heat) required to allow the water phase transition from liquid to vapor. As a consequence, this energy does not contribute to the increase in temperature in the tissue. It is worth noting that the radial dimensions of the area delimited by isothermal contour at 55 °C in the adrenal gland (Figure 7) differ less than 0.5 mm between the two numerical scenarios. This small variation is nearly unchanged both at 30 W and 60 W. 

Overall, the vaporization model has a negligible impact on the final extent of the ablation zones for the settings considered. Instead, water vaporization shows a sizable impact on the maximum temperature reached at the end of the MWA procedure. 

The model of water vaporization influences not only the maximum temperature achieved, but also the trend of an increase in temperature over time, especially at small distances from the applicator. At 4 mm from the antenna and at 30 W, the temperature increases with an approximately linear slope of 4 °C·s^−1^ for the first 20 s for both numerical models. From 20 s onwards, the temperature in the case of the standard BHE continues to increase steadily at a rate of approximately 3 °C·s^−1^ (Figure 8A). In the case of modified BHE, temperature elevations slow to a rate of approximately 1 °C·s^−1^ for the next 10 s and a slower rate of 0.1 °C·s^−1^ from 10 s to 60 s (Figure 8B). At the same distance (4 mm) from the applicator and at 60 W, the approximately linear slope at which the temperature increases is 7 °C·s^−1^. This rate is similar between the two models (i.e., standard BHE and modified BHE) for the first 10 s only. When the vaporization model is considered, the increment in temperature slows to 3 °C·s^−1^ between 10 s and 35 s. Then, such a slope rate becomes much slower (0.08 °C·s^−1^) up to the end of the procedure. It can be also observed that at a further distance from the applicator (7 mm), the water vaporization is less influential due to the lower temperatures reached as compared with the 4 mm distance. 

In summary, on the one hand, the results of this study show that the water vaporization model has a high impact on the maximum temperature reached at the end of the MWA, especially in proximity to the applicator. The same model also influences the trend of a temperature increase with time. On the other hand, the vaporization model shows a negligible effect on the extent of the ablation zone, for the power and time settings considered (Figure 7). Finally, it is worth noting that a dynamic simulation requires a higher computational load as compared with standard numerical simulations. 

## 4. Conclusions

This study investigates the effect of MWA operating at 5.8 GHz on the ability of the fat layer to shield the electromagnetic energy and create asymmetric ablation zones in the adrenal gland. 

The results of the study show that the ‘side firing’ approach is capable of creating asymmetric ablation zones at 2.45 GHz as shown in the previous studies, and also at 5.8 GHz, using consistent power and time settings. 

Because of the higher value in effective conductivity of the adrenal cortex at 5.8 GHz as compared with 2.45 GHz, a faster deposition of the electromagnetic energy in the tissue is expected. The results of this study show that the faster interaction between the tissue and the electric field helps to achieve larger ablation zones. These ablation zones are able to cover adreno-cortical adenomas of 10 mm minimum diameter. 

Finally, in this study, we show that the unrealistically high temperatures observed in the tissue during the ablation can be compensated, accounting for the vaporization of the tissue water content in the numerical studies. This is particularly true when the temperatures exceed 70 °C. In addition, the vaporization model has no sizable influence on the prediction of the extent of the ablation zone. 

## Figures and Tables

**Table 1:** Tissue dielectric and thermal properties at 25 °C employed in the numerical simulations. Relative permittivity (ε_r_) and effective conductivity (*σ* (S·m^−1^)) of cortex and medulla at 5.8 GHz refer to the mean values of dielectric data reported in [11].

Parameter	Fat	Cortex	Medulla
Relative permittivity, *ε_r_*	8.3	41.2	47.6
Effective conductivity, *σ* (S·m^−1^)	0.5	4.2	5.1
Specific heat capacity, *c* (kJ·m^−3^·K^−1^)	2.3	3.6	3.7
Thermal conductivity, *k* (W·m^−1^·K^−1^)	0.2	0.5	0.5
Density, *ρ* (kg·m^−3^)	911	1025	1025

**Table 2:** Summary of the power and time settings and of actual positions of the fiber optic sensors with reference to the feed of the antenna for each MWA experimental procedure. The original positions of the sensors were 4 mm and 7 mm along the radial distance from the axis of the applicator both in the adrenal tissue and surrounding fat.

Sample #	Settings		Sensors Actual Position 1		Sensors Actual Position 2	
	Power (W)	Time (s)	Fat	Adrenal Gland	Fat	Adrenal Gland
1	30	60	4 mm	5 mm	7 mm	7 mm
2	30	60	4 mm	4 mm	6 mm	6 mm
3	30	60	5 mm	6 mm	8 mm	6 mm
4	60	60	3 mm	4 mm	6 mm	6 mm
5	60	60	4 mm	4 mm	7 mm	6 mm
6	60	60	5 mm	5 mm	8 mm	8 mm

**Table 3:** Radial and longitudinal extents calculated at 30 W (first line) and at 60 W (second line) in the simplified geometry and in the 3D model, in the case of 5.8 GHz. The radial and the longitudinal dimensions of ablation zones measured experimentally are reported in terms of mean and standard deviation for each power and time setting considered.

Settings				Simplified Model		3D Model Orientation #1				3D Model Orientation #2				Experimental	
P (W)	t (s)	Fat (mm)		Adrenal Gland (mm)		Fat (mm)		Adrenal Gland (mm)		Fat (mm)		Adrenal Gland (mm)		Adrenal Gland (mm)	
		R	L	R	L	R	L	R	L	R	L	R	L	R	L
30	60	4	8	9	15	5	11	8	11	5	9	9	16	8 ± 2	10 ± 2
60	60	7	15	10	20	7	17	10	15	7	14	10	20	9 ± 2	13 ± 2

## Data Availability

All data are included in the article.
